# Roles for mHealth to support Community Health Workers addressing COVID-19

**DOI:** 10.1177/1757975920967924

**Published:** 2020-11-11

**Authors:** James O’Donovan, Rebecca Hamala, Margaret Nalubwama, Mathew Ameniko, George Govina, Nowai Gray, Raj Panjabi, Daniel Palazuelos, Allan Saul Namanda

**Affiliations:** 1 Department of Education, Learning and New Technologies Research Group, University of Oxford, United Kingdom; 2 Division of Research and Health Equity, Omni Med, Uganda; 3 Community Health Water and Sanitation Agency, Ghana; 4 Last Mile Health, Liberia; 5 Harvard Medical School, United States of America; 6 Division of Global Health Equity, Brigham and Women’s Hospital, United States of America; 7 Department of Global Health and Social Medicine, Harvard Medical School, United States of America; 8 Partners in Health, United States of America

**Keywords:** Community Health Workers, COVID-19, coronavirus, mHealth, mobile technologies

Given many healthcare systems around the world are buckling under the weight of COVID-19, the pandemic has presented significant challenges to countries with weak healthcare systems (1). One cadre of healthcare workers at the forefront of addressing the pandemic are Community Health Workers (CHWs) (2), who have roles in prevention, detection and response. The term ‘Community Health Worker’ encompasses a wide range of lay-healthcare workers and the precise nomenclature used to describe CHWs varies from region to region (3); however, a widely accepted definition proposed by the World Health Organization (WHO) is that they ‘should be members of the communities where they work, should be selected by the communities, should be answerable to the communities for their activities, should be supported by the health system but not necessarily a part of its organization, and have shorter training than professional workers’ (4). Importantly, CHWs do not include ‘professional’ facility-based health workers such as doctors and nurses, or allied-healthcare professionals, such as physiotherapists or medical assistants. Similarly, the exact work of CHWs varies globally; however, they generally deliver healthcare services in the community focused on integrated maternal and child health services (5), but more recently their scope of work has increased to include services focused on non-communicable diseases (6). Their work is also wide ranging and not only focuses on disease prevention and management, but also on ‘community development activities, referrals, recordkeeping and collection of data on vital event(s)’ (4).

With the increasing use of mobile technologies (mHealth) to support CHWs in their work-related duties (7), we suggest how mHealth could support CHWs at this challenging time. To contextualise this commentary, it is important to note smartphone ownership is increasing in low- and middle-income countries and by 2025 the Global System for Mobile Communications estimates that 83 million people in Sub-Saharan Africa (over 40% of the population) will be mobile internet subscribers (8). Similarly, smartphone ownership is becoming more prevalent among CHWs. For example, a recent study from Malawi estimated that 50% of CHWs own a smartphone (9). Likewise, many non-governmental organisations are partnering with ministries of health to support and improve health system-strengthening initiatives. For example, in Uganda, Living Goods equips over 2000 CHWs with smartphones (10), and in Liberia 100% of CHWs supported by Last Mile Health have a smartphone (11).

In this article we document three roles for mHealth to support CHWs in addressing the current COVID-19 pandemic, including (a) facilitating case recording and contact tracing; (b) education, training and supervision; and (c) facilitating communication. This is written from the perspective of CHWs and CHW programme coordinators in Ghana, Liberia, Mexico and Uganda as we deal with the current COVID-19 pandemic.

## Facilitating case recording and contact tracing

The first potential role for mHealth is in facilitating CHWs, supervised and equipped with appropriate personal protective equipment, to record and log cases, as well as to support contact tracing, an approach that was adopted during the Ebola epidemic of 2014–16 in West Africa (12, 13). In relation to the current COVID-19 pandemic, a team at the University of Oxford has created a mobile application (app) to facilitate case recording and contact tracing (14). When positive cases are confirmed, a series of SMS messages are sent to individuals in the relevant geographic area, advising them to take necessary precautions such as self-isolating, thus slowing the spread of the disease. These messages could also be sent to CHWs so they are kept updated on positive cases in the areas they are responsible for and take appropriate action as required. If such an approach were to be adopted and used by CHWs, it would be important the data are sent to a secure, centralised, national database to avoid a disjointed approach towards disease surveillance and monitoring.

Existing apps that normally survey individuals for symptoms of non-communicable disease such as diabetes and hypertension are also being modified in countries such as Bangladesh to canvas individuals at the community level for symptoms related to COVID-19 (15). mHealth is therefore one way to facilitate remote triage and assessment. It could also help alleviate the immense personal risk that CHWs are taking on, as well as reduce the chances of CHWs becoming inadvertent vectors of viral spread. By collecting self-reported data on symptomatology remotely, mHealth could allow for real-time mapping and the prediction of potential spread of COVID-19. This is critical in countries where health systems are already stretched thin by helping ensure that scarce resources can be directed to targeted areas where they are needed most.

## Education, training and supervision

The second role for mHealth is for CHW education, training and supervision. Given that COVID-19 is a novel virus, it is important that CHWs receive accurate, culturally appropriate, and relevant information to help them recognise, treat and prevent potential cases (especially as guidelines and protocols change rapidly in this ever-evolving pandemic). One platform that CHWs can access through their mobile phones is the WHO COVID-19 online training resource (16). This is available for free and has been translated into multiple languages. Furthermore, as social distancing begins to take place and CHWs are unable to attend in-person training sessions, remote training and supervision facilitated through mHealth initiatives will become increasingly important. For instance, in Liberia, the Ministry of Health is working with partners to incorporate COVID-19 content in its Community Health Academy learning management system, which is accessed by over 3500 CHWs and supervisors via their smartphones (17). These resources may also be used to facilitate community-based learning, such as sharing training videos with the local population.

## Facilitating communication

**Figure 1.**
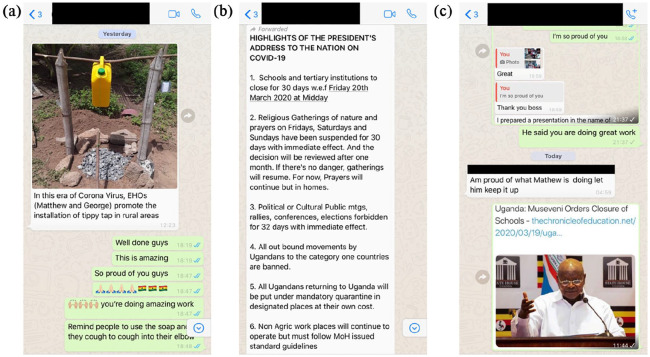
WhatsApp messages exchanged between Community Health Workers (CHWs) in Ghana and Uganda. (a) Sharing best practices regarding construction of low-cost handwashing facilities; (b) sharing up-to-date information regarding government policies; and (c) messages of support and encouragement (Credit and permissions: Margaret Nalubwama, Mathew Ameniko, Allan Saul Namanda).

The third role for mHealth is to facilitate communication between CHWs. CHWs in Uganda and Ghana have already established collaborative groups via mobile-messaging apps, such as WhatsApp, to share messages highlighting preventative measures (e.g. the construction of hand-washing stations, Figure 1(a)) and up-to-date information regarding government policies (Figure 1(b)). Messages of encouragement and praise have also boosted CHWs’ morale as they begin to practice social distancing and have provided peer-to-peer support during this challenging time (Figure 1(c)). The WHO COVID-19 WhatsApp messaging platform, released on 20 March, has also been a welcome addition. This automated messaging system delivers accurate messages regarding COVID-19 symptoms, up-to-date epidemiological data, and the latest news (18).

### Challenges of mHealth

It is important to caveat that the use of mHealth is not without its challenges. The risks include disjointed approaches towards implementation, misinformation being spread by group messaging platforms, privacy and data concerns, and technical difficulties for CHWs in remote and rural areas where cellular network coverage is poor (19). It is important those responsible for designing, implementing and managing such mHealth initiatives at this time are aware of these limitations and act upon them accordingly.

For example, from an immediate, practical perspective, one approach to address connectivity issues would be to identify a central hub with a stable fixed router (e.g. at a local health centre) where CHWs could visit and upload data to a central server. One limitation of such an approach, however, is the risk that CHWs would take in traveling to the health centre if there is community spread. It is also important to note the issue of unreliable and poor cellular connectivity is a complex, longstanding and ongoing problem, which requires addressing at a government level. For example, the governments of India, Rwanda, South Africa and Uganda have all convened advisory committees to strengthen the enabling environment for mobile health-facilitated care delivery, such as improved cellular infrastructure in remote regions (20). Similarly, by partnering with national and local government agencies it is more likely that mHealth initiatives can contribute to an overall health-systems strengthening approach.

Other solutions to the above challenges include promoting the use of regulated channels of information, such as The WHO WhatsApp information group, to increase information quality assurance. It will also be important to involve key local stakeholders, such as the CHWs themselves, in the design of such programmes. This participatory approach can help designers uncover and address local barriers to adoption, which can in turn facilitate uptake of such programmes and increase local relevance and acceptability (21).

## Conclusion

The specific ways in which mHealth approaches can support CHWs will vary from country to country. In some locations where CHWs are tasked with conducting household testing, mHealth will play an important role in facilitating them with case detection, such as through suspected case logging. In other locations mHealth might have an important role in facilitating CHWs who are responsible for notifying individuals with confirmed COVID-19. Whatever role mHealth has, it is important to consider how it can be integrated in a coordinated manner to a country’s COVID-19 response and that it contributes to an overall health system strengthening approach.

With the potential limitations (and proposed solutions) to mHealth initiatives that we have outlined taken into consideration, we strongly encourage other CHWs and CHW organisations to begin to explore how mHealth approaches can be leveraged to address and lighten the burden COVID-19 has placed on already strained healthcare systems around the world.
